# ﻿Two new *Halamphora* (Bacillariophyta) species from the marine coasts off Livingston Island, Antarctica

**DOI:** 10.3897/phytokeys.195.81632

**Published:** 2022-05-11

**Authors:** Ralitsa Zidarova, Plamen Ivanov, Nina Dzhembekova, Myriam de Haan, Bart Van de Vijver

**Affiliations:** 1 Institute of Oceanology at the Bulgarian Academy of Sciences, 40 Parvi May Str., 9000 Varna, Bulgaria Institute of Oceanology at the Bulgarian Academy of Sciences Varna Bulgaria; 2 Institute of Biodiversity and Ecosystem Research at the Bulgarian Academy of Sciences, 2 Mayor Yurii Gagarin Str., 1113 Sofia, Bulgaria Institute of Biodiversity and Ecosystem Research at the Bulgarian Academy of Sciences Sofia Bulgaria; 3 Meise Botanic Garden, Nieuwelaan 38, B-1860, Meise, Belgium Meise Botanic Garden Meise Belgium; 4 University of Antwerp, Department of Biology – ECOSPHERE, Universiteitsplein 1, B-2610 Wilrijk, Belgium University of Antwerp Wilrijk Belgium

**Keywords:** *Amphora* sensu lato, diatoms, marine benthos, taxonomy

## Abstract

During a survey of the marine benthic diatom flora on the coasts off Livingston Island (South Shetland Islands, Maritime Antarctic Region), two *Halamphora* species that could not be identified based on the currently available literature, were observed. Detailed light and scanning electron microscopy observations and thorough comparison with similar taxa in the literature revealed that both taxa should be described as new species. The first taxon, *Halamphorakenderoviana***sp. nov.**, was most likely misidentified in past Antarctic studies, and included within the range of another taxon, *Halamphoracoffeaeformis*. Analysis of literature data showed that the second new taxon, *Halamphoramoncheviana***sp. nov.**, has been previously reported from the Antarctic Continent (but as an unidentified species). The new taxa are compared with similar *Halamphora* taxa worldwide. Data on their ecology and distribution are also provided.

## ﻿Introduction

In the past two decades considerable effort has been undertaken to improve our understanding of the diversity, species identities and distribution of the terrestrial and freshwater diatoms (Bacillariophyta) in the Antarctic realm (Zidarova et al. 2016 and references therein). In contrast, the marine benthic diatom flora of the region remained far less studied. Although in the past 30 years three new marine benthic diatom genera have been described from islands in the Southern Ocean [*Tabulariopsis* D.M.Williams (Williams 1988), *Brandinia* L.F.Fernandes (in Fernandes et al. 2007), and *Australoneis* J.M.Guerrero & Riaux-Gob. (in Guerrero et al. 2021)], and several new species have been recognized and described within existing genera [including *Berkeleya* Grev. (Medlin 1990), *Cocconeis* Ehrenb. (Al-Handal et al. 2008, 2010), *Gomphonemopsis* Medlin (Al-Handal et al. 2018), *Nitzschia* Hassall (Al-Handal et al. 2019), *Melosira* C.Agardh (Fernandes and de Souza-Mosimann 2001), *Pteroncola* R.W.Holmes & Croll (Almandoz et al. 2014) and *Rhoicosphenia* Grunow (Ligowski et al. 2014)], a recent study on the marine benthic diatom flora from South Bay (Livingston Island, South Shetland Islands) showed that a large number of the recorded taxa could not be identified with certainty up to species level, with some of these taxa most likely being new to science (Zidarova et al. 2022).

The present paper describes two new marine species in the genus *Halamphora* (Cleve) Levkov, observed in several recently collected samples from the coasts off Livingston Island, part of the archipelago of the South Shetland Islands (Maritime Antarctic Region).

The genus *Halamphora* (Cleve) Levkov, originally described in 1895 by Cleve as a subgenus of *Amphora* Ehrenb. ex Kütz., was raised to genus level in 2009 (Levkov 2009). The characteristic features of the genus include a moderately to strongly dorsiventral valve outline, an eccentric raphe system, uni- to biseriate striae composed of round to elliptical and even transapically elongated areolae, internally occluded by hymenes, and a girdle composed of numerous open copulae. *Halamphora* species can be found both in freshwater and marine ecosystems (Levkov 2009). In the Antarctic Region, Van de Vijver et al. (2014) revised the freshwater *Halamphora* species, describing several new taxa, but in the marine realm data on *Halamphora* (or former *Amphora*) species are scarce. One of the observed species in the present study has most likely been misidentified and reported in the earlier Antarctic literature under the name of another, presumably widespread marine *Halamphora* species, *H.coffeaeformis* (C.Agardh) Kütz., whereas the second taxon is unknown. Following extensive light and scanning electron microscopy observations and comparisons of their morphology with similar taxa from all over the world and from the Antarctic Region, both taxa are described as new species: *Halamphorakenderoviana* sp. nov. and *Halamphoramoncheviana* sp. nov. A survey of the Antarctic literature with iconographic material provided additional information on their basic ecology and Antarctic distribution.

## ﻿Materials and methods

**Figure 1.**
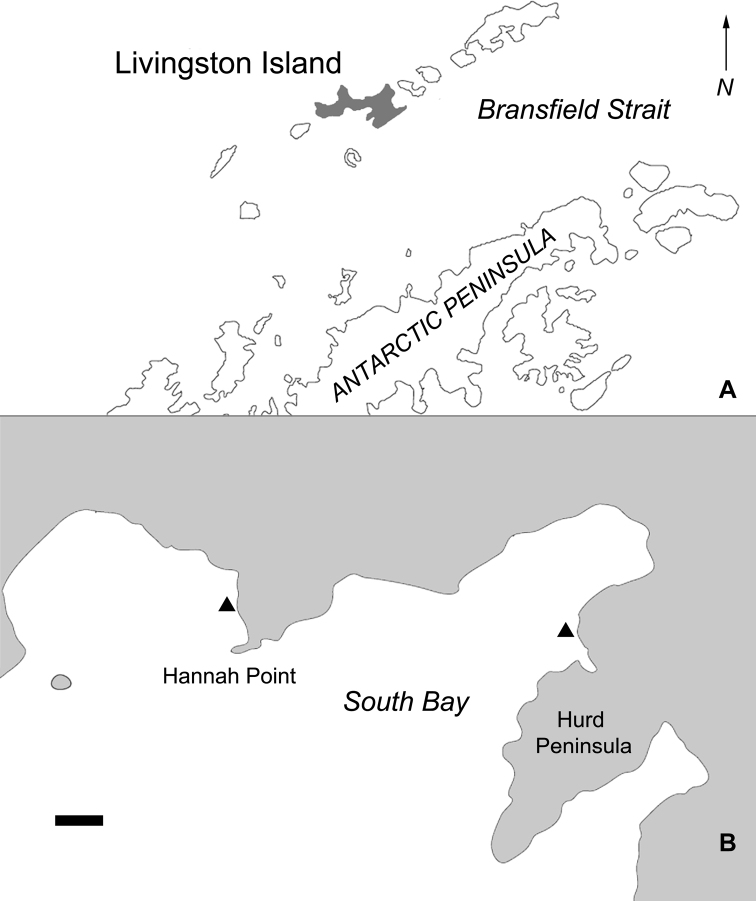
Map showing the position of Livingston Island relative to the Antarctic Peninsula (**A**), and the locations of the sampling sites (**B**, triangles). Map outlines are based on OpenStreetMap contributors (www.openstreetmap.org), edited and arranged using Adobe Illustrator and Adobe Photoshop. Scale bar: 2 km (**B**).

Livingston Island is the second largest of the South Shetland Islands, located ca. 130 km north of the Antarctic Peninsula (Fig. 1). Samples were collected in December 2018 and February 2020 from the epilithon of small pools on coastal rocks at the southern coasts of the island, including Hannah Point area and the eastern shores of South Bay (Fig. 1). These pools are small water basins, formed during low tide on or between larger rocks on the coasts, and having variable water temperature and salinity levels (Zidarova et al. 2022 and references therein). The biofilm covering the bottom or sides of the pools was collected using a toothbrush and preserved with 3% formaldehyde *in situ*. Samples, with their environmental parameters measured with a handheld multi-parameter meter WTW3410 during sample collection, are listed in Table 1.

**Table 1.** Samples containing the new taxa, with their environmental parameters.

Sample	Date	pH	Salinity, PSU	Conductivity, mS/cm	О_2_, %	О_2_, mg.L^-1^	Water Т, ^О^C
MO’	21/12/2018	8.4	33.1	52.2	n/a	n/a	5.5
LT10	04/02/2020	n/a	6.5	11.5	126.0	11.6	18.8

For light microscopy (LM), diatom samples were prepared following the method of Hasle and Fryxell (1970). Cleaned material was mounted in Naphrax. LM observations were conducted using an Olympus BX51 light microscope at 1000× magnification (N.A. 1.30), equipped with Differential Interference Contrast (DIC) optics and Olympus digital imaging system. For scanning electron microscopy (SEM), part of the suspension was filtered through 5 μm Isopore polycarbonate membrane filters (Merck Millipore), after air-drying pieces of which were affixed with double sided carbon stickers (Agar Scientific Carbon Tabs) on 12.7 mm Ø aluminium stubs (Agar Scientific Ltd), coated with a platinum layer of 20 nm and studied using a JEOL-JSM-7100F field emission scanning electron microscope at 2 kV. Slides and stubs are stored at the BR-collection (Meise Botanic Garden, Belgium). Plates with the microphotographs of the species were prepared using Adobe Photoshop. For stria number, measurements were done starting from the valve middle. Terminology for taxa descriptions follows Round et al. (1990), Levkov (2009), and Stepanek and Kociolek (2018). In a search for more ecological and distributional data for the taxa we describe, a survey of the earlier Antarctic literature was conducted, including the major older works (e.g. Van Heurck 1909; Peragallo 1921; Frenguelli and Orlando 1958; Simonsen 1992), as well as more recent reports with iconographic material, such as Roberts and McMinn (1999), Cremer et al. (2003), and others.

## ﻿Results

### ﻿Descriptions of new species

Systematics follows the adopted in DiatomBase (Kociolek et al. 2022)

**Phylum Bacillariophyta Haeckel**

**Class Bacillariophyceae Haeckel**

**Family Amphipleuraceae Grunow**

#### Genus *Halamphora* (Cleve) Levkov

##### ﻿ Halamphora kenderoviana

Taxon classificationPlantaeNaviculalesAmphipleuraceae

Zidarova, P.Ivanov, Dzhembekova, M.de Haan & Van de Vijver sp. nov.

55756560-6844-5EE2-9343-68965B35BA9C

Fig. 2A–M

###### Holotype.

**Figure 2.**
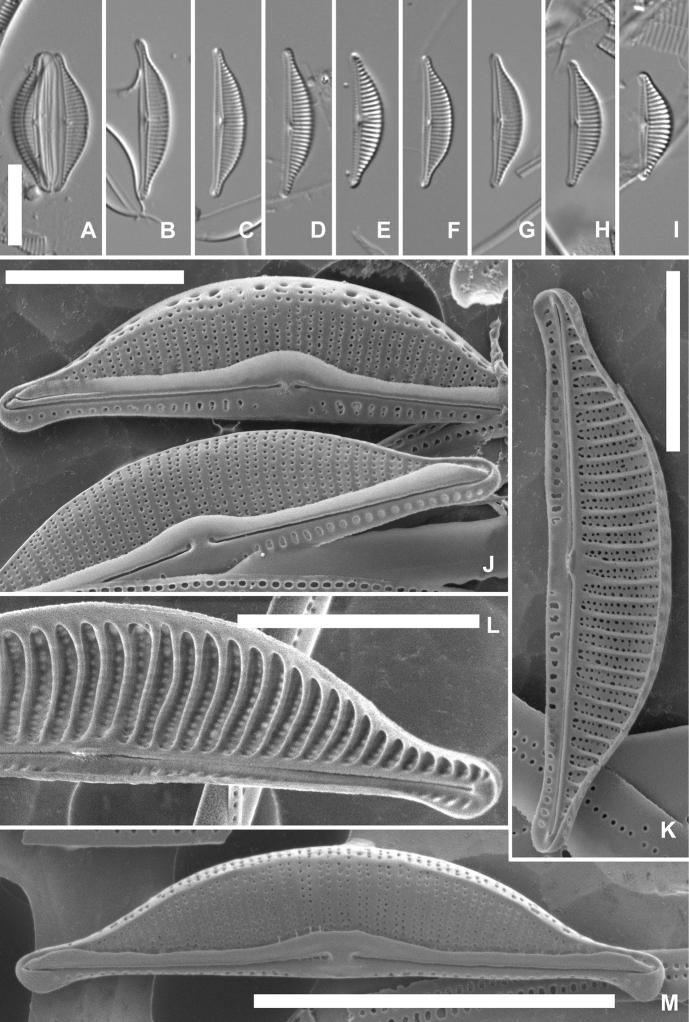
*Halamphorakenderoviana* sp. nov., valves from the type population from South Bay, Livingston Island **A–I** valves under LM, with fig. A showing an entire frustule **D** represents the holotype **J** two valves under SEM externally with details of the central raphe endings and mantle areolae **K**SEM of an entire valve internally **L**SEM of a valve internally, showing the prominent raised costae between the striae and the internal areolar occlusions **M**SEM of an entire valve externally, with a view on the mantle and the dorsal ridge. Scale bars: 10 µm (**A–I, M**); 5 µm (**J–L**).

Slide BR-4681, Fig. 2D represents the holotype, Meise Botanic Garden, Belgium. PhycoBank (http://phycobank.org/103140).

###### Isotype.

Slide 401, University of Antwerp, Belgium.

###### Type locality.

Antarctica, Livingston Island, South Bay, Mongolian (Reserve) Port, small pool on a coastal rock during low tide, epilithon. 62°38'50"S, 60°22'26"W. Sample MO’, *leg.* R. Zidarova, coll. date 21 Dec. 2018.

###### Description.

***LM description*** (Fig. 2A–I). Valves semi-lanceolate to narrowly semi-elliptic, with a straight ventral and distinctly convex dorsal margin. Valve dimensions (n = 27): length 13.5–20.5 µm, width 3.5–4.5 µm. Apices slightly ventrally bent, in larger valves protracted, rostrate to almost subcapitate (Fig. 2B), in smaller valves weakly protracted, subrostrate (Fig. 2F–I). Raphe branches straight. Central raphe endings expanded, slightly dorsally bent (Fig. 2A, C, I). Distal raphe fissures not discernible in LM. Axial area narrow. Central area on the dorsal side very small to usually absent, on the ventral side clearly enlarged. Dorsal striae parallel to weakly radiate in the middle, becoming more radiate towards the apices, 18–20 in 10 µm. Occasionally, one or two striae in the valve middle shortened (Fig. 2E, G), forming a very small dorsal central area. Ventral striae discernible in LM, interrupted in the valve middle (Fig. 2C–F, I), 27–28 in 10 µm.

***SEM description*** (Fig. 2J–M). Externally, valves possess a distinct raphe ledge, running along the entire length of the valve, clearly widened in the valve middle, truncated and slightly expanded at the apices (Fig. 2J, M). Central raphe endings positioned relatively close together, slightly bent towards the dorsal side and pore-like enlarged (Fig. 2J, M). Terminal raphe fissures hooked to the dorsal side (Fig. 2J, M). Dorsal striae biseriate, composed of rounded poroids, the latter 60–65 in 10 µm. Striae continuing on the mantle, following a narrow dorsal ridge (Fig. 2M), where often reduced to a single, large areola (Fig. 2J). Striae on the ventral side short, composed of only one or two rounded areolae, often fused to form a single elongated areola (Fig. 2J). Internally, central raphe endings terminating onto a fused central helictoglossa (Fig. 2K). Terminal raphe endings finishing onto small helictoglossae (Fig. 2K). Striae internally located between narrow, quite prominently raised virgae (costae). Areolae internally occluded by individual hymenes (Fig. 2L).

###### Etymology.

The new species is named after our colleague Dr Lyubomir Kenderov, hydrobiologist at the Faculty of Biology, University of Sofia, with whom RZ shared two Antarctic seasons, and who was often a helping hand during field work in Antarctica.

###### Ecology, Antarctic distribution and associated diatom flora.

*Halamphorakenderoviana* was typically observed in tidal pools (Zidarova et al. 2022, as *Amphora* sp.5), but only found in abundance (17.5% of the counted valves) in the type locality, a tidal pool with alkaline water and a salinity level of 33.1 PSU (Table 1). Other common taxa in the sample are *Parlibellusrhombicus* W.Greg., *Tabulariopsisaustralis* (Perag.) D.M.Williams, and several *Navicula* species, including N.aff.perminuta Grunow and *N.glaciei* Van Heurck. So far, *H.kenderoviana* is known to be present with certainty on the marine coasts of the South Shetland Islands (Livingston Island). Earlier, Cremer et al. (2003) reported a very similar taxon as *Amphoracoffeaeformis* (Cremer et al. 2003, fig. 13) from sediment cores in Windmill Island, East Antarctica. SEM observations will be needed to confirm that it is conspecific with *H.kenderoviana*, but it seems likely that at least some of the records of *A.coffeaeformis*, transferred to *Halamphora* by Levkov (2009) as *H.coffeaeformis* (Kütz.) Levkov, from saline waters in Antarctica might represent *H.kenderoviana*.

##### ﻿ Halamphora moncheviana

Taxon classificationPlantaeNaviculalesAmphipleuraceae

Zidarova, P.Ivanov, Dzhembekova, M.de Haan & Van de Vijver sp. nov.

9B24CBB1-5846-53C5-A0B1-45A3BFDABE06

Fig. 3A–M

###### Holotype.

**Figure 3.**
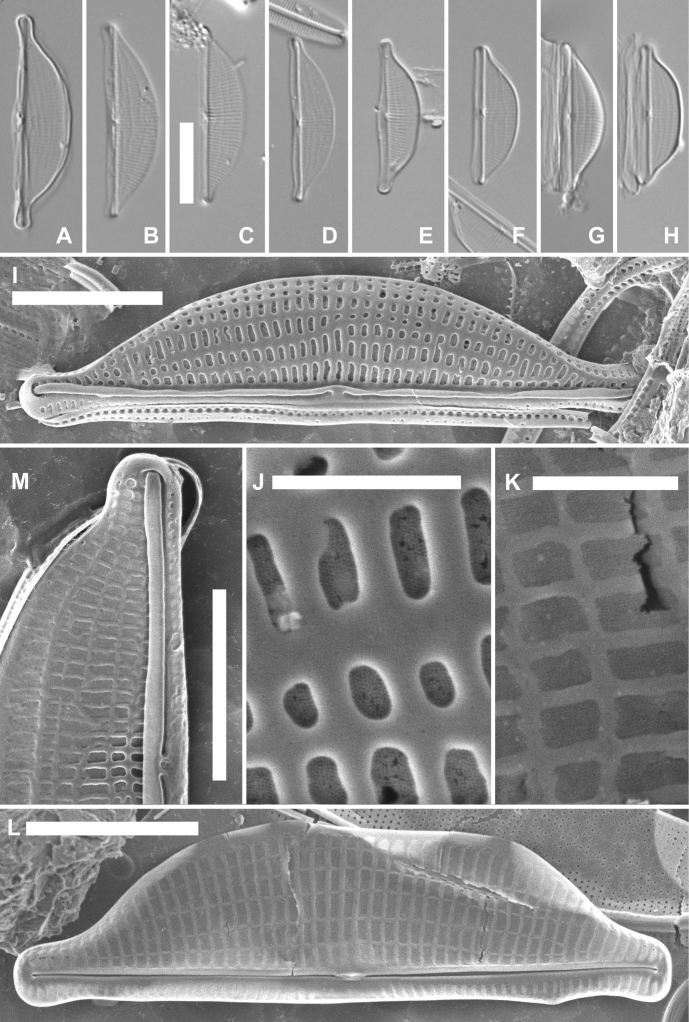
*Halamphoramoncheviana* sp. nov., valves from the type population from South Bay **A–H**LM views of several valves **G** represents the holotype **I**SEM of an entire valve externally, showing the dorsal striae and the raphe endings **J**SEM, detail of the areolae externally, showing the recessed porous foramina **K**SEM, detail of the striae and areolae internally, showing the porous internal areolar foramina **L**SEM of an entire valve internally **M**SEM, external view of a valve with areolae arranged in longitudinal lines, most likely in a state of development. Scale bars: 10 µm (**A–H**); 5 µm (**I, L, M**); 1 µm (**J, K**).

Slide BR-4682, Fig. 3G represents the holotype, Meise Botanic Garden, Belgium. PhycoBank (http://phycobank.org/103141).

###### Isotype.

Slide 400, University of Antwerp, Belgium.

###### Type locality.

Antarctica, Livingston Island, Hannah Point, small pool on a coastal rock north of the penguin rookeries, epilithon. 62°38'30"S, 60°36'32"W. Sample LT10, *leg.* R. Zidarova, coll. date 04 Feb. 2020.

###### Description.

***LM description*** (Fig. 3A–H). Valves weakly silicified, broadly semi-elliptic, with a more or less straight ventral and distinctly convex dorsal margin. Apices protracted, subcapitate in larger valves (Fig. 3A), becoming only weakly protracted, rostrate in smaller valves (Fig. 3F–G). Valve dimensions (n = 23): length 16.0–27.5 µm, width 5.0–7.0 µm. Raphe straight. Central raphe endings straight, enlarged (Fig. 3A, F–H). Terminal raphe fissures not discernible in LM. Axial area narrow, central area absent. Dorsal striae parallel to weakly radiate in the middle, becoming more radiate towards the apices, 24–27 in 10 µm, crossed by several undulating longitudinal lines (Fig. 3A–H).

***SEM description*** (Fig. 3I–M). Externally, valves show a narrow, but distinct raphe ledge, slightly elevated and running on the entire length of the valve (Fig. 3I, M). Central raphe endings relatively close together, weakly dorsally bent, indistinct (Fig. 3M) to weakly enlarged (Fig. 3I). Terminal raphe fissures shortly hooked to the dorsal side (Fig. 3I, M). Dorsal striae on the valve face composed of usually 3–5 transapically elongated, sometimes almost rectangular areolae with recessed finely porous foramina (Fig. 3J). Areolae forming longitudinal rows (Fig. 3I, M). On the mantle, areolae get smaller (Fig. 3I). Distinct marginal dorsal ridge lacking (Fig. 3I, M). Internally, central raphe endings terminating onto fused helictoglossae. Terminal raphe endings finishing onto small helictoglossae (Fig. 3L). Areolae internally rectangular, arranged in regular transverse and longitudinal rows between raised virgae and vimines, possessing finely porous recessed foramina (Fig. 3K, L). Ventral striae only internally observed on the valve face, 33–34 in 10 µm, composed of a single elongated areola (Fig. 3L).

###### Etymology.

The new species is named after Prof Dr Snejana Moncheva, phycologist and former Director of the Institute of Oceanology at the Bulgarian Academy of Sciences, to thank her for considering our (RZ, NDzh) employment and career possibilities at the Institute.

###### Ecology, Antarctic distribution and associated diatom flora.

*Halamphoramoncheviana* was most abundant in the epilithon of a small coastal pool, having a relatively low salinity (6.5 PSU, sample LT10, Table 1), where it was found together with *Craspedostauroslaevissimus* (W.West & G.S.West) Sabbe and several *Nitzschia*, *Melosira* and *Navicula* species. Roberts and McMinn (1999, Pl. 1, figs 10–11) recorded the same taxon as *Amphora* sp. d from the Vestfold Hills on the Antarctic Continent, although their reported valves were slightly larger (length 30–35 µm, width 5–8 µm) and with a slightly coarser striation of “approximately” 22 striae in 10 µm. Nevertheless, the SEM photo of the species, identified as *Amphora* sp. d in Roberts and McMinn (1999, plate 1, fig. 11), presenting a valve externally with striae, composed of a few transapically elongated areolae on the dorsal side and forming irregular longitudinal lines on the valve face, confirms the conspecificity between the species observed on the Antarctic Continent, and *H.moncheviana*. Roberts and McMinn (1999) reported the species from hypersaline lakes. Based on their and our findings, *H.mocheviana* is apparently a very tolerant species to changes in salinity. Likely the same taxon was also depicted by Priddle and Belcher (1981, fig. 3l, as *Amphora* sp.), which they observed in the epilithon of a large, shallow pool (Pool 7) situated near the sea, together with several species of marine origin, including *Craspedostauroslaevissimus* (reported as *Tropidoneislaevissima* W.West & G.S.West).

## ﻿Discussion

Based on the observed set of morphological features of *H.kenderoviana* and *H.moncheviana*, both these species from the coasts of Livingston Island clearly belong to the genus *Halamphora*, as defined in Levkov (2009). The morphological analysis also showed that the combination of features in both taxa is sufficiently unique to justify their description as new species.

*Halamphorakenderoviana* is one of the many *Halamphora* species, having a valve outline with protracted apices and biseriate striae, similarly to *Halamphoracoffeaeformis* and its related taxa (Stepanek and Kociolek 2018). The morphology of *H.coffeaeformis*, an often-misinterpreted species, was studied in detail by Archibald and Schoeman (1984), and later discussed in Levkov (2009) and in Stepanek and Kociolek (2018). In contrast to *H.coffeaeformis*, where the biseriate dorsal striae continuing onto the mantle are interrupted by more or less developed dorsal ridge, the mantle striae in *H.kenderoviana* are often reduced to only a single enlarged areola after the narrow dorsal ridge. Moreover, the biseriate striae in *H.coffeaeformis* are composed of very small (fine) and closely positioned areolae (see for instance figs 102, 105 and 108 in Archibald and Schoeman 1984, and Pl. 46 in Stepanek and Kociolek 2018), compared to the relatively large and distantly spaced areolae in the dorsal striae of *H.kenderoviana* (Fig. 2J). In *H.coffeaeformis* the two rows of areolae in each stria are also positioned very close together (e.g. figs 108, 116, 121, 142, 155, etc., in Archibald and Schoeman 1984, and Pl. 46, figs 6, 7 in Stepanek and Kociolek 2018), leaving wide virgae between the striae, whereas the two rows of areolae in the biseriate striae in *H.kenderoviana* are clearly widely spaced, leaving narrower virgae between the striae (e.g. Fig. 2J, M). *Halamphoracoffeaeformis* is also a larger taxon, with a width of usually above 5 µm, and all populations, considered to be identical with the type, show a more or less arched raphe (e.g. Pl. 91, figs 1–14 in Levkov 2009 and Pl. 45, figs 1–8 in Stepanek and Kociolek 2018). Two other *Halamphora* species with biseriate striae, *H.aponina* (Kütz.) Levkov and *H.isumiensis* Stepanek et al. possess fine, rounded areolae, with biseriate striae continuing onto the mantle (Levkov 2009, Pl. 233, figs 2, 7 and Stepanek and Kociolek 2018, Pl. 53, fig. 1, respectively). *Halamphoraaponina* has slightly longer valves (> 23 µm) with a slightly finer striation of 20–22 striae in 10 µm, and an almost linear raphe ledge, only expanded near the apices (Levkov 2009), but not in the valve middle, whereas *H.isumiensis* has a much finer striation on the ventral side (ca. 36 striae in 10 µm vs. 27–28 in *H.kenderoviana*), and a very distinct dorsal marginal ridge (Stepanek and Kociolek 2018), not observed in *H.kenderoviana*. *Halamphorakenderoviana* also differs from all the above-mentioned species with the presence of prominently raised, though narrow internal costae.

Kellogg and Kellogg (2002), who compiled a list of diatom taxa, recorded in the Antarctic by the year 2000, listed *Halamphoracoffeaeformis* and some (earlier considered) infraspecific taxa of the latter, showing a similar valve outline and comparable valve dimensions to *H.kenderoviana*, such as Amphoracoffeaeformisvar.borealis (Kütz.) Cleve. Amphoracoffeaeformisvar.borealis is treated as a synonym of *A.borealis* Kütz. (see Kellogg and Kellogg 2002, p. 71 and references therein), a species now transferred to *Halamphora* as *H.borealis* (Kütz.) Levkov (Levkov 2009). However, *H.borealis* has uniseriate dorsal striae (Levkov 2009), contrary to the biseriate striae in *H.kenderoviana*. Other, and more recently described taxa, similar in valve outline and possessing biseriate dorsal striae (as both *H.coffeaeformis* and *H.kenderoviana*), include *H.bistriata* Stepanek & Kociolek (Stepanek and Kociolek 2018), *H.tumida* (Hustedt) Levkov (Sar et al. 2004; Levkov 2009) and *H.americana* Kociolek (Kociolek et al. 2014). When compared to *H.kenderoviana*, they all generally have a larger valve width of above 4 µm and biseriate striae in only part of the valve face dorsally.

The most similar taxa in LM in terms of valve outline and striation pattern include *Halamphoranagumoi* Stepanek et al. and *H.banzuensis* Stepanek et al. However, under SEM, *H.nagumoi*, described from the Pacific coasts, presents very closely positioned central raphe endings and a prominent dorsal ridge (Stepanek and Kociolek 2018, Pl. 55, fig. 1), contrary to *H.kenderoviana*, lacking these features. *Halamphorabanzuensis* from the Banzu flat near Tokyo (Japan) has a much finer striation on the ventral side (40–43 vs. 27–28 striae in 10 µm in *H.kenderoviana*), a well-developed dorsal ridge, and striae dorsally are separated by raised virgae on the valve exterior (Stepanek and Kociolek 2018, Pl. 70, figs 3, 4), features not present in *H.kenderoviana*. Finally, based on valve outline and striation, *Amphoracognata* Cholnoky is also morphologically similar, but the latter has a slightly finer striation on the dorsal side (22–24 striae vs. 18–20 striae in 10 µm in *H.kenderoviana*), and a slightly larger width (5–6 µm vs. up to 4.5 µm in *H.kenderoviana*) (Cholnoky 1966). As SEM observations are lacking for *A.cognata*, it is not possible to compare the ultrastructure of both species at present. They, however, differ in ecology. *Amphoracognata* was described from warm springs in South Africa (Cholnoky 1966), a very different habitat, compared to the cold-water Antarctic marine coasts where *H.kenderoviana* was discovered, and it is rather unlikely that the two taxa are conspecific. The Argentinean Amphora (Halamphora) capitellata Freng., whose original description and drawing are provided by Sar et al. (2009), is larger (valve width 5–6 µm), and based on the drawing in Sar et al. (2009, p. 49), possesses a less convex dorsal side and a small central area on the dorsal side, contrary to *H.kenderoviana*.

*Halamphoramoncheviana*, showing internally rectangular areolar openings arranged in regular rows, and externally irregular longitudinal lines on the dorsal side in LM, can hardly be confused with any other *Amphora* or *Halamphora* taxa. In valve outline, with its shortly protracted subcapitate apices, it bears only a slight resemblance to the South American brackish species *Halamphoramira* (Krasske) Levkov. The latter is a much larger taxon, with a length exceeding 35 µm, a width above 9 µm, and with a strongly arched raphe (Lange-Bertalot et al. 1996, as *Amphoramira* Krasske; Levkov 2009), contrary to *H.moncheviana*. The smaller *H.miroides* Levkov also presents a strongly arched raphe, and finely punctate striae. Moreover, it is a typically freshwater (and not marine) species, known mostly from Africa (Levkov 2009). Both the Antarctic *Halamphoralateantarctica* Van de Vijver et al. and *H.vyvermaniana* Van de Vijver et al. possess a distinct arched raphe, and a distinct dorsal marginal ridge (Van de Vijver et al. 2014), in contrast to *H.moncheviana*. Moreover, they both lack the internal stria structure of *H.moncheviana*, composed of rectangular areolae, arranged in regular rows (e.g. Van de Vijver et al. 2014, fig. 9K and fig.11E). Amphora (Halamphora) eunotia Cleve var.striolata Freng. from Argentina has larger valves with a much coarser striation of 14–18 striae in 10 µm (Sar et al. 2009). *Halamphorasiqueirosii* López-Fuerte et al. from hypersaline waters in Mexico has semi-lanceolate valves with a cut in the raphe ledge in the valve middle (López-Fuerte et al. 2020), a feature not observed in *H.moncheviana*, and lacks the arrangement of the elongated areolae in irregular longitudinal rows on the dorsal side externally. The freshwater species *Halamphoracoloradiana* Stepanek & Kociolek is a smaller taxon, with a width of only 2.5–4.5 µm (vs. > 5 µm in *H.moncheviana*), with a very dense striation near the apices (> 29 striae in 10 µm), and lacks the clearly rectangular internal areolar openings (Stepanek and Kociolek 2013, fig. 85), present in *H.moncheviana*. Finally, the valves shown on the original drawing of *Amphorakuehniae* Schoeman in the diatom collection files of the Academy of Natural Sciences, Philadelphia (Potapova et al. 2022), show some similarity to *H.moncheviana* in their striation pattern; however, the valves from Lesotho, which were examined and depicted under LM by Levkov (2009), who also transferred the species to the genus *Halamphora* (as *H.kuehniae* (Schoeman) Levkov), differ from *H.moncheviana* with their strongly dorsiventral valves with coarse, but more rounded and not arranged in longitudinal rows areolae. *Halamphorakuehniae* is also a much larger taxon with an arched (and not straight) raphe (Levkov 2009).

Two other marine taxa, *Amphoraantarctica* Hust. (Hustedt 1958) and *A.barrei* Manguin (Manguin 1960), were described from Antarctica. They share a similar valve outline with strongly protracted apices, overlapping dimensions and a very fine striation pattern, suggesting they might be conspecific, as noted earlier by Hargraves (1968). Both, however, clearly differ from *H.moncheviana* based on their dense and almost indiscernible striation pattern in LM and the presence of much more protracted apices (see also figs 5, 6 in Cremer et al. 2003 for *A.antarctica*), excluding all conspecificity.

## Supplementary Material

XML Treatment for Halamphora kenderoviana

XML Treatment for Halamphora moncheviana
